# Surrogate-Assisted Fine Particulate Matter Exposure Assessment in an Underground Subway Station

**DOI:** 10.3390/ijerph19042295

**Published:** 2022-02-17

**Authors:** Liyang Liu, Hui Liu, Yiming Ma

**Affiliations:** 1 School of Architecture and Urban Planning, Huazhong University of Science and Technology, Wuhan 430074, China; liuliyang-199577@163.com; 2 Hubei Engineering and Technology Research Center of Urbanization, Huazhong University of Science and Technology, Wuhan 430074, China; 3 State Key Laboratory of Advanced Electromagnetic Engineering and Technology, School of Electrical and Electronic Engineering, Huazhong University of Science and Technology, Wuhan 430074, China

**Keywords:** computational fluid dynamics, particulate matter exposure, pedestrian flow analysis, surrogate model

## Abstract

With the increase in subway travelers, the air quality of underground enclosed spaces at subway stations has attracted much more attention. The study of pollutants exposure assessment, especially fine particulate matter, is important in both pollutant control and metro station design. In this paper, combining pedestrian flow analysis (PFA) and computational fluid dynamics (CFD) simulations, a novel surrogate-assisted particulate matter exposure assessment method is proposed, in which PFA is used to analyze the spatial-temporal movement characteristics of pedestrians to simultaneously consider the location and value of the pedestrian particulate generation source and their exposure streamline to particulate matter; the CFD model is used to analyze the airflow field and particulate matter concentration field in detail. To comprehensively consider the differences in the spatial concentration distribution of particulate matter caused by the time-varying characteristics of the airflow organization state in subway stations, surrogate models reflecting the nonlinear relationship between simulated and measured data are trained to perform accurate pedestrian exposure calculations. The actual measurement data proves the validity of the simulation and calculation methods, and the difference between the calculated and experimental values of the exposure is only about 5%.

## 1. Introduction

With the progress of economic construction and the increase of population density, subways have become one of the most important transportation modes. However, with the dramatic increase in passenger flow and urban air pollution problems, the air quality and population health problems caused by various pollutants in underground subway stations deserve extensive attention [1,2]. Subways, together with residences and workplaces, constitute three important pollution-exposed microenvironments for urban populations [3]. Considering that the subway is mostly built underground, with poor air mobility and high pedestrian density, its air pollution problem is more serious compared to the latter two locations. Various types of particulate matter have been identified as one of the major air pollutants in the subways, those with a particle size less than 2.5 μm (PM2.5) can even enter the alveoli through the lower respiratory tract, thus causing respiratory diseases [4] and have been proven to be an important vector for viruses such as COVID-19 [5]. Therefore, research on the fine particulate matter pollution in subway stations is of great importance in the construction of subway stations, pollutant control, and health risk assessment.

In recent years, studies on air pollution in subway stations have gradually increased. Initially, scholars started with the chemical composition of pollutants and focused on the measurement of different pollutant concentrations, including gaseous pollutants, particulate pollutants, and microorganisms [6,7]. Then, the effects of various types of equipment in subway stations on the distribution of pollutants were also included in the study. For example, Kim et al. analyzed the effects of platform screen door (PSD) systems and ventilation systems on the distribution characteristics of pollutants [8], Lee et al. analyzed the effect of the ventilation system on indoor air quality and developed a prediction model between ventilation demand and indoor air quality [9]. However, most of the studies were conducted on completed subway stations and analyzed through field studies and actual measurements of pollutant concentrations, which are not conducive to taking air pollution into account in the design and construction of subway stations. Some scholars have tried to apply numerical simulation methods, such as computational fluid dynamics (CFD), to the study of pollutant distribution characteristics [10]. A. Bolourchi et al. conducted CFD simulations of the Iman Khomeini subway station in Tehran to analyze particulate matter concentration levels and propose optimization measures to meet the respiratory health needs of workers and passengers [11]. Teodosiu et al. used CFD methods to determine whether the ventilation efficiency of the subway station under emergency evacuation conditions can ensure that passengers are not exposed to high temperatures during evacuation or high concentrations of carbon monoxide disturbance [12]. Song et al. simulated the spatial distribution characteristics of particulate matter in the long underground passageway of the Shanghai South Railway Station using the CFD method [13]. However, how to fully consider the time-varying characteristics of the airflow organization state in the subway station when applying CFD methods is still an issue to be further studied.

Most of the work listed above has been carried out on the concentration or spatial distribution characteristics of the pollutants themselves in the subway station space without really studying the effects of pollutants on pedestrians, i.e., without taking the behavioral characteristics of pedestrians into account for the assessment of their exposure to pollutants. Pedestrians are not only the source of pollutants in enclosed spaces but also the ultimate target of pollutant-related studies, so it is very important to take the behavioral characteristics of pedestrians into account. The concept and steps of exposure assessment are explained in detail in literature [14,15] published by the United States Environmental Protection Agency, with the two most important steps being the determination of pollutant concentrations and the identification of exposure pathways. The main difference from previous studies is the consideration of spatial-temporal characteristics of pedestrians. Related studies have been carried out in both indoor and outdoor spaces [16,17,18], but they are mostly based on statistical methods, which are strongly correlated with the specificity of the scene, lack generality, and have less application in underground subway stations.

To deal with the above problem and facilitate the accurate air pollution exposure assessment in underground subway stations, this paper proposes a novel particulate matter exposure assessment method that combines the pedestrian flow analysis (PFA) and the CFD method.PM2.5, one of the most harmful air pollutants, is selected as the research object. The PFA is used to analyze the exposure paths of pedestrians in subway stations and give the value and location of pedestrian particulate matter generation sources, while the CFD simulation is used to analyze the spatial concentration distribution characteristics of particulate matter. Furthermore, for the subway stations with PSDs, the opening and closing of PSDs bring two different airflow organization states, and the particle generation sources are different in the two states, so steady-state field CFD simulations are needed. To integrate the particulate matter distribution under two airflow organization states in the exposure assessment, the simulation results of steady-state fields under two airflow organization states and the actual measurement results of particulate matter concentration distribution are used to train surrogate models of simulated and measured values by support vector machine (SVR) and *K*-means algorithms, and finally combine the PFA results to achieve an accurate particulate matter exposure assessment. All simulation results are verified with actual measurement data.

## 2. Materials and Methods

### 2.1. Exposure Calculation

Pollutant exposure refers to the process of human exposure to a certain concentration of a pollutant over a certain period of time, regardless of inhalation or exhalation processes. Exposure is an important indicator for conducting exposure assessment [14], which integrates the effects of pollutant concentration, exposure time, and human exposure parameters, and can accurately reflect the relationship between pollutants and human health effects [19].

Exposure can be calculated using the following equation,

(1) $$E=\int_{T}C(t)dt$$

where *E* denotes the exposure value; *C*(*t*) represents the concentration of pollutants in the environment to which the human body is exposed at time *t*, referred to as the exposure concentration; *T* denotes the duration of exposure.

However, when the time scale is large, such as when the timing units of more than hours are used, or when computational simplification is required, Equation (1) can be of the following form. This method is commonly used in the relevant literature to calculate the exposure, and in some literature only the average concentration in the environment is focused on [16,17].

(2) $$E=\sum_{i=1}^{N}\mathrm{avg}(C_{i})•T_{i}$$

where *avg*(*C_i_*) denotes in the *i*th microenvironment, the average concentration of pollutants in period *T_i_*; *T_i_* indicates the duration of exposure in the *i*th microenvironment. The main difference between Equations (1) and (2) is that the former takes into account the variation of pollutant concentrations over time and space, while the latter does not, so the former is more accurate since it has a smaller calculation step.

### 2.2. Type Selection of the Studied Subway Station

To ensure the generality and applicability of this study, a classification of subway stations from the perspective of particulate matter exposure assessment is needed first to select the most suitable station for the example study. This study is conducted in Wuhan, which is one of the mega-cities in China with a large population and many subway lines. As of January 2021, Wuhan had nine subway lines in operation, with a total of 240 subway stations, a total length of over 600 km, and an average daily passenger flow of more than three million people. This section will classify the subway stations in Wuhan from the following four perspectives to select an appropriate station for the subsequent study.

- Perspective 1: Air environment and ventilation conditions. This classification perspective corresponds to whether the station is an underground station or an elevated station. Compared with elevated stations, underground stations are more enclosed, and the ventilation conditions are much worse. It is difficult to discharge pollutants, so the underground stations will be chosen as the research object. The subway stations in Wuhan are also mainly underground, accounting for 79.1% of the total stations.
- Perspective 2: Space utilization rate of the station platform. This classification perspective is based on whether the type of platform is an island platform or a side platform. Island stations are smaller in size and have higher space utilization rate than side stations, which need to serve passengers in two directions. Although island platforms improve space utilization, passengers are more likely to congregate, which leads to greater exposure of passengers to pollutants. Therefore, the stations with an island platform will be chosen as the research object. There are 41 side-platform stations and 199 island-platform stations in Wuhan, and the percentage of island platforms is as high as 82.9%.
- Perspective 3: Airflow organization mode and pollutant sources. This classification perspective is based on whether the station adopts the PSD system. PSDs will change the airflow organization mode and can also greatly prevent pollutants from the tunnels from entering the platforms. For safety reasons, the majority of stations in China use PSD systems, and Wuhan is no exception, with all underground stations equipped with PSD systems. To ensure generality, the stations with PSD systems will be used for this study.
- Perspective 4: The matching degree of the design passenger flow with the actual passenger flow. The design of a subway station will make a preliminary estimate of its passenger flow; the form of train formation has a direct relationship with the design passenger flow, therefore, the design passenger flow will also affect the subway station space. However, with the development of the city, some of the early built stations are designed for small passenger flows but ended up assuming much larger passenger flows. It leads to a high density of passengers in the station, serious crowd gathering, and greater exposure to air pollution. In Wuhan, the trains on the earlier built lines 2, 3, and 4 are all in six-carriage formations, which means that each train has six carriages; they are designed for small passenger flows. The stations on the three lines have smaller station spaces but carry the majority of the city’s passenger traffic. In contrast, new lines 7, 8, and 11 are designed with eight-carriage formations; they have large station spaces but less passenger traffic. To account for this imbalance, the stations on lines 2, 3, and 4 will be used for this study.

**Figure 1:**
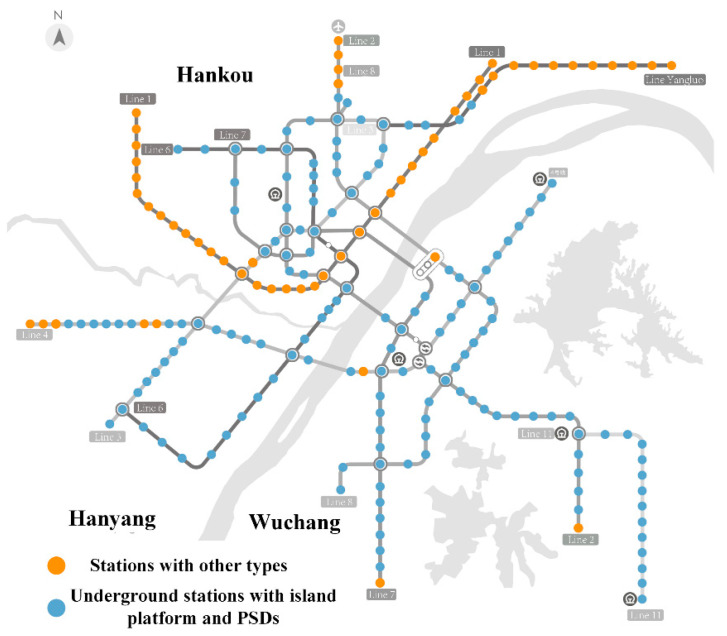
Underground stations with island platforms and PSD systems.

Based on the above analysis, an underground stations on lines 2, 3, or 4 with an island platform and the PSD system will be selected for study in this paper. Figure 1 gives the distribution of underground island stations with PSD systems in Wuhan, which accounts for 76.2% of the total. Therefore, the study’s generality and adaptability can be ensured.

### 2.3. Experimental Site Description

**Figure 2:**
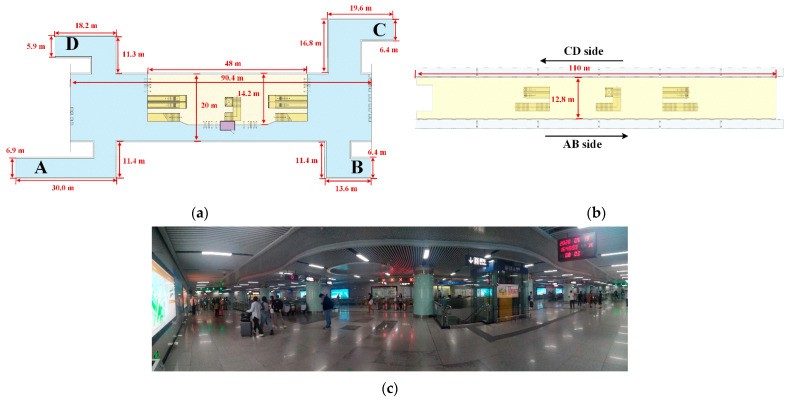
The floor plan of the concourse floor and platform floor: (**a**) The concourse floor; (**b**) The platform floor; (**c**) A view of the concourse floor.

Combining the above selection criteria, the Sports Center Station on Line 3 of the Wuhan Metro is selected as the research object to illustrate the proposed method. This station has two spacious floors, the concourse floor and the platform floor, with a total area of 13,940 m^2^; the concourse floor has four exits with a height of 3.5 m, the platform floor is an island platform with the PSD system, the height of the platform is 3 m. More detailed parameters are given in Figure 2. The station has no interchange or other transportation attributes, and there are no connected underground businesses, with easy access to ground-level slow-moving traffic, and the vast majority of people entering the station are passengers.

Taking this station as an example, in the following section, the PFA model based on the Massmotion software and the CFD simulation model based on Ansys Fluent is given, and the numerical simulations are performed. A novel surrogate-assisted exposure calculation method is proposed and used to accurately calculate the PM2.5 exposure during boarding and the alighting of pedestrians. The simulation results and exposure calculation results are compared with the experimental data to validate the proposed method.

## 3. Simulation Model

### 3.1. PFA Model

**Figure 3:**
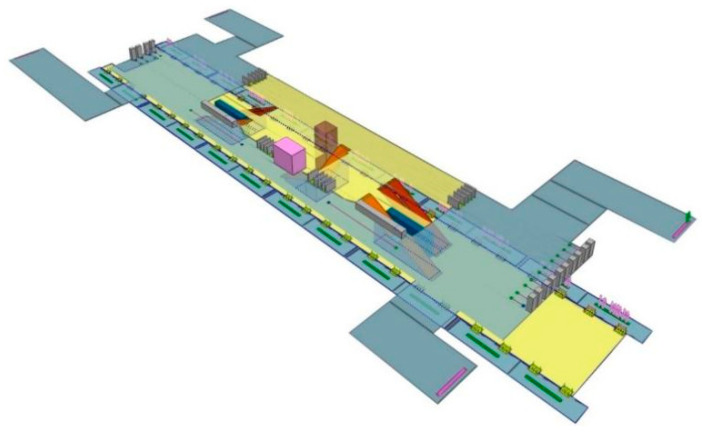
The concourse floor in the Massmotion model.

PFA is performed using the Massmotion software based on the social force model; the effectiveness of this software had been demonstrated in [20,21]. According to the floor plan of this station, The PFA model was built according to the floor plan of this station. Figure 3 shows the PFA model of the concourse floor.

The following boundary conditions of this PFA model need to be artificially given. It should be noted in advance that, to reduce the difficulty of field measurements, and considering that metro operators do not allow measurements to be carried out during peak traffic hours, the measured data in this paper were obtained during off-peak hours.

Firstly, the walking speed of pedestrians in the subway station should be given. This speed data is obtained in reference to the videos, according to the method given in [13]. The mean speed of pedestrians is set to 1.25 m/s with a variance of 0.2, and it is considered to be following a normal distribution.

Secondly, pedestrian preferences for the four exits need to be determined, as well as the behavioral choices of pedestrians entering the subway station. Table 1 gives statistical information on the data of the pedestrians entering the subway station from the four exits during one off-peak hour in the morning and one in the afternoon during a weekday on Wednesday, 11 November 2020. Eighty percent of pedestrians use their cards to enter the station, while 20% of pedestrians go to the ticket machine to buy tickets and then enter the station, disregarding the few pedestrians who pass through the subway station to cross the street. The ratio of pedestrian choice for the four exits A, B, C, D is set to 3.5: 2: 1: 3.5. 

Finally, the software also needs to give the number of boarding and alighting pedestrians on each train in different directions. Table 2 shows the statistics of pedestrians’ information during one weekday (11 November 2020) morning off-peak hour; the interval between trains is 6 min.

Pedestrians’ choice of action path in the MassMotion simulation takes into account action path length, queuing time, and facility usage. The path is chosen based on the perceived cost of all available routes so that pedestrians reach their final goal without backtracking, as follows,

(3) $$C\mathrm{ost}=(W_{D}\times (\frac{D_{G}}{V}))+(W_{q}\times Q)+(W_{L}\times L)$$

where total route cost (*Cost*) denotes the total travel time along the route (s); *W_D_* is the “distance” weight; *D_G_* is the distance from the person’s position to the final target (m); *V* is the person’s speed (m/s); *W_q_* is the “queue” weight; *Q* is the expected time in the queue before reaching the link entrance (s); *W_L_* is the “geometric component” weight; *L* is the geometric component type cost (s), for this case, the geometric component type can be specifically divided into escalators and stairs. For more details about these settings, one can refer to [21].

### 3.2. CFD Model

Airpak3.0 and ANSYS Fluent19.0 together form a CFD simulation model, in which the former is used to build a subway station model and mesh division while solving the continuous-phase airflow field; while the latter is used to simulate the particle concentration field based on the Airpak model, and the particles in the simulation are selected as PM2.5. On the choice of the numerical simulation model, the standard *κ-ε* turbulence model is used to simulate airflows and turbulence in this semi-closed building, which can be described as follows [22],

(4) $$\frac{\partial \left(\rho k\right)}{\partial t}+\frac{\partial \left(\rho k\mu_{i}\right)}{\partial x_{i}}=\frac{\partial }{\partial x_{j}}[\left(\mu +\frac{\mu_{1}}{\sigma_{\kappa }}\right)\frac{\partial \kappa }{\partial x_{j}}]+G_{\kappa }+G_{b}-\rho \varepsilon$$

(5) $$\frac{\partial \left(\rho \varepsilon \right)}{\partial t}+\frac{\partial \left(\rho \varepsilon \mu_{i}\right)}{\partial x_{i}}=\frac{\partial }{\partial x_{j}}[\left(\mu +\frac{\mu_{1}}{\sigma_{\varepsilon }}\right)\frac{\partial \varepsilon }{\partial x_{j}}]+G_{1\varepsilon }\frac{\varepsilon }{\kappa }(G_{k}{+C}_{3\varepsilon }G_{b})-C_{2\varepsilon }\rho \frac{\varepsilon^{2}}{k}$$

where *κ* and *ε* denote kinetic energy of turbulence flow and the rate of turbulent dissipation transport, respectively; *ρ*, *t*, and *μ_i_* denote the density, time, and the velocity of flow, respectively; *μ* is kinetic viscosity; *G_k_* is turbulence production item; *G_b_* is the turbulent kinetic energy generation caused by Buoyancy; *σ_k_* and *σ_ε_* are the turbulence coefficients, *σ_k_* = 1.0, *σ_ε_* = 1.3; *Cμ* = 0.09; *C*_1*ε*_ = 1.44, *C*_2*ε*_ = 1.92, and *C*_3*ε*_ = 1.44.

Meanwhile, considering that the volume fraction of PM2.5 is less than 10% in air, the discrete phase model (DPM) based on the Euler-Lagrange equation is used to perform a gas-solid two-phase flow simulation. Finally, the following assumptions are given:

- The air is isotropic;
- The PM2.5 filtration efficiency by the primary filter of the ventilation system is 40%;
- Consider the effect of continuous relative to discrete phase particle population and ignore the effect of discrete relative to continuous phase;
- Disregarding the adsorption of particulate matter by pedestrians. Resuspension of settled particulate matter due to pedestrian movement is also ignored;
- When the PSDs are closed, the amount of air leakage from the PSD is negligible.

**Figure 4:**
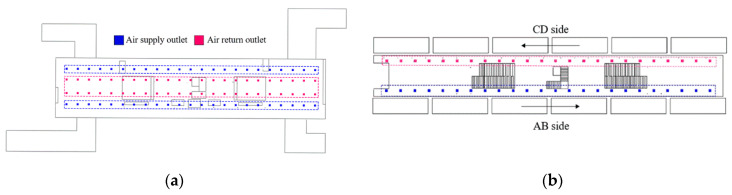
Arrangements of supply and return air outlets in the two floors: (**a**) The concourse floor; (**b**) The platform floor.

**Figure 5:**
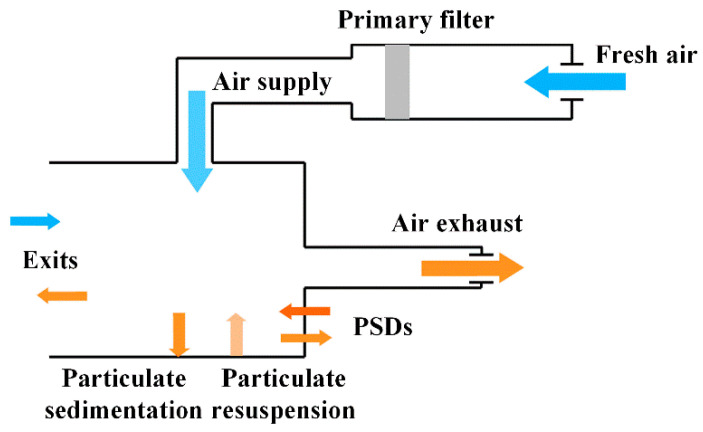
Simplified diagram of ventilation in the station during the non-air-conditioning season.

The modeling dimensions of the public area are 90.4 m × 20 m × 3.5 m (the concourse floor) and 110 m × 12.8 m × 3 m (the platform floor). The arrangements of supply and return air outlets in the two floors are shown in Figure 4, the air outlet sizes of the concourse floor and platform floor are 300 mm × 300 mm and 600 mm × 500 mm, respectively. This study is conducted during the non-air-conditioning season, so the outdoor temperature is lower than the supply air temperature, the outdoor air is sent directly to the concourse floor and platform floor after passing through the primary filter without cooling treatment, while the return air is all discharged to the outdoors, as shown in Figure 5 [23].

**Figure 6:**
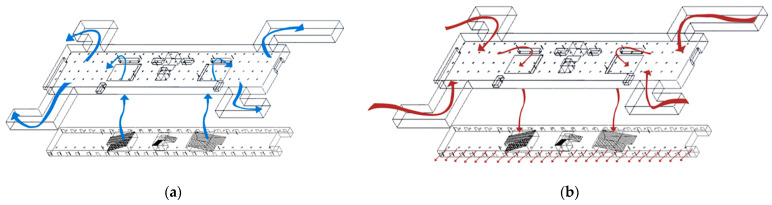
Two main airflow organization states: (**a**) PSDs closed; (**b**) PSDs opened.

The airflow organization pattern in the station can be simplified to the following two states [23,24], one is the PSDs closed state (Figure 6a); when the train does not arrive, if the air leakage of PSDs are neglected, PSDs are well-sealed and the air inside the subway flows to the outdoor through the exits. The particulate matter generated in the tunnel by the train running and rubbing the track will not enter the platform [23]; the other one is the PSDs opened state (Figure 6b), since the low air pressure in the tunnel and carriages, the platform air flows to the tunnel and carriages. Meanwhile, the outdoor air enters the concourse floor. When PSDs are closed, the sources of particulate are air supply outlets, equipment, and pedestrians; but when the PSDs are opened, outdoor air enters so that the exits are also particulate sources, therefore, the two airflow organization states need to be simulated separately.

Table 3 summarizes the boundary condition settings of this CFD simulation model, including the concourse/platform floor air supply velocity *v_c_*/*v_p_*, the value of lighting load *W_l_*, the particulate density *ρ_p_* and diameter *D_p_*, the outdoor particulate matter concentration *μ_out_* and air supply outlets particulate matter concentration *μ_sup_*, and particle generation rates for pedestrians *r_pe_* and equipment *r_eq_*. The literature [25] states that the particle generation rate for a single person in an enclosed space ranges from 6.5 to 15.2 mg/h, and the mean value of 10 mg/h is taken in this paper.

**Figure 7:**
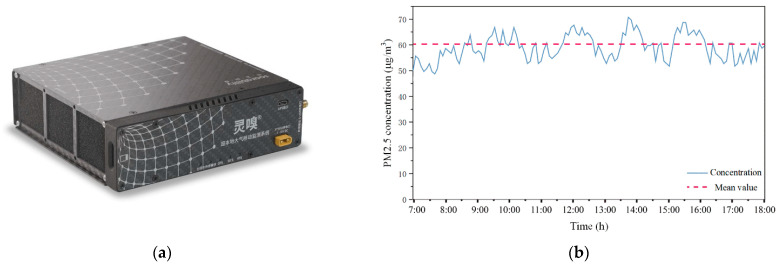
Sniffer 4D and Outdoor PM2.5 concentration measurement results: (**a**) Sniffer 4D instrument; (**b**) Measurement results of outdoor PM2.5 concentrations on 11 November 2020.

In Table 3, the particulate concentration at air supply outlets is set as the outdoor particulate concentration multiplied by the PM2.5 filtration efficiency of the ventilation system (40%), and the particulate concentration at the outlet is equal to the outdoor particulate concentration. The outdoor PM2.5 concentration is measured by the Sniffer 4D instrument (Soarability Technologies, Inc., Shenzhen, China) [26], as shown in Figure 7a; This instrument has a detection sensitivity of 0.1 μg/m^3^ for PM2.5 concentration measurement with a time interval of 1 s. Figure 7b shows the measurement results of outdoor PM2.5 concentrations at Exit A on 11 November 2020. The measurement points of outdoor PM2.5 concentration were arranged at the four exits and the wind pavilion of the station. Since there are no complex commercial buildings near the station, the terrain is gentle and the traffic flow is moderate, the daily average PM2.5 concentrations at the five measurement points were relatively consistent, about 61.5 μg/m^3^.

## 4. Simulation Results and Exposure Calculation

### 4.1. PFA Results

There are two main purposes of conducting PFA simulations. One is to obtain pedestrian exposure streamlines and the other is to obtain the locations and values of pedestrian particulate generation sources to more accurately model the spatial distribution characteristics of particulate matter.

**Figure 8:**
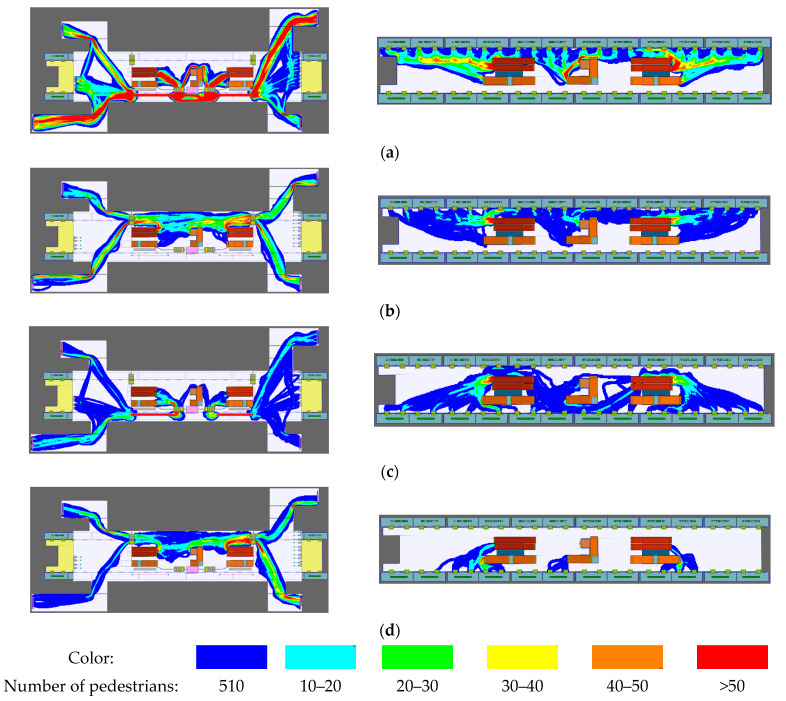
Space utilization analysis results for boarding and alighting pedestrians in different directions: (**a**) CD side boarding; (**b**) CD side alighting; (**c**) AB side boarding; (**d**) AB side alighting.

For the first purpose, the movement track of each pedestrian is available in the software. Meanwhile, the space utilization analysis of different directions of boarding and alighting pedestrians for the station’s concourse and platform floors can also be performed, which indicates the pedestrian’s choice of path. Figure 8 shows the space utilization analysis results for each subway trip. The closer the area to red, the higher the space utilization and the more concentrated the flow of pedestrians.

For the second purpose, the spatial density of pedestrians in the subway station needs to be analyzed to determine the crowded areas and the congestion of pedestrians. The spatial average density characterizes the average number of pedestrians per unit time and unit area. Higher density values indicate more severe pedestrian congregation in this area, and therefore mean that it is more prone to congestion, and larger values for pedestrian particulate generation sources. The average density of pedestrians can be characterized by the service level of the area, which can be found in the International Air Transport Association (IATA) waiting level of service comparison table given in Massmotion software [21], as shown in Table 4.

**Figure 9:**
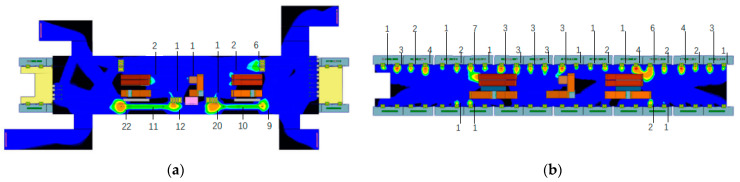
Simulation results of pedestrian spatial density at two floors: (**a**) Concourse floor; (**b**) Platform floor.

The simulation results of pedestrian spatial density at the station concourse floor and platform floor are shown in Figure 9a,b, respectively, while the average number of pedestrians in the area below service level B is also marked in the figure. The location of the pedestrian particulate generation source is set at the center of the circle used to calculate the density, and the value is determined by multiplying the number of people by the amount of particulate generated by a single person. The congested areas in the concourse floor are mainly at the inbound security check queue, in front of the inbound gates, and in front of the outbound gates, corresponding to service levels E, E, and C, respectively. The congested areas on the platform floor are mainly the stairway entrance and the area in front of the PSDs.

**Figure 10:**
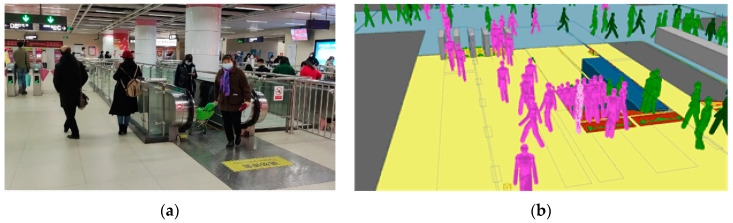
Observed and simulated pedestrian distribution at the stairway of the concourse floor: (**a**) Observed result; (**b**) Simulation result.

**Figure 11:**
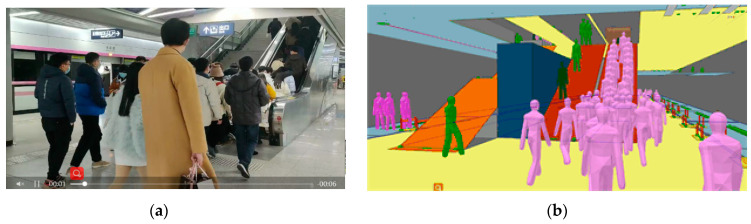
Observed and simulated pedestrian distribution at the stairway of the platform floor: (**a**) Observed result; (**b**) Simulation result.

Figure 10 and Figure 11 give comparisons of the observed and simulated pedestrian distribution in part of the crowded areas at the station concourse and platform to validate the PFA model. Observations of pedestrian flow were made during the same period as the field measurement results.

### 4.2. CFD Results

**Figure 12:**
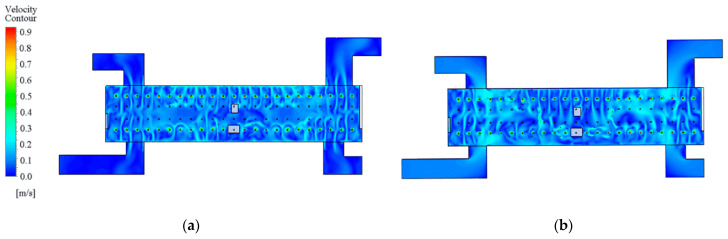
Simulation results of the airflow field at the station concourse floor under different airflow organization: (**a**) PSDs closed; (**b**) PSDs opened.

**Figure 13:**
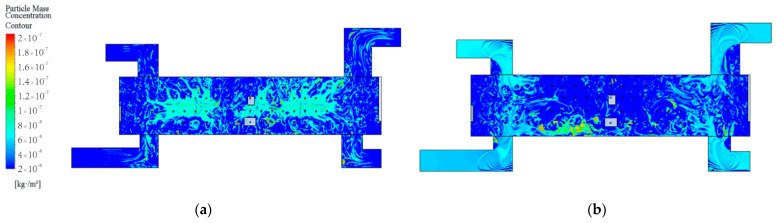
Simulation results of particle mass concentration contour at the station concourse floor under different airflow organization: (**a**) PSDs closed; (**b**) PSDs opened.

**Figure 14:**
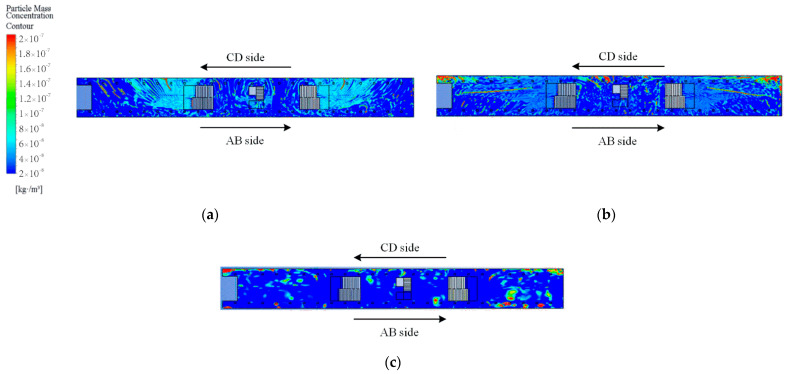
Simulation results of particle mass concentration contour at the station platform floor under different airflow organization: (**a**) AB side PSDs opened; (**b**) CD side PSDs opened; (**c**) PSDs closed.

After determining the locations and values of pedestrian particulate generation sources, CFD simulations can further be performed to analyze the spatial distribution characteristics of the particulate matter. The CFD model is solved in two steps; the first step is to solve the airflow field without the particulate matter source. For example, Figure 12 shows simulation results of the airflow field at the station concourse floor under different airflow organizations. After that, the particulate matter source is then set and the DPM model is used to complete the solution of the particulate matter concentration field. The spatial concentration distribution of PM2.5 at the breathing plane (Height = 1.5 m) with different airflow organization is obtained as shown in Figure 13 and Figure 14.

For the concourse floor, the PM2.5 is mainly concentrated at the inbound security checkpoint, inbound vending machines, and outbound vending machines, and at the stairway entrance and the return air outlet where the crowd gathers. The main reason is that, firstly, pedestrians are one of the main PM2.5 sources, so the concentration of PM2.5 is larger where pedestrians gather; secondly, PM2.5 from the platform floor can move to the concourse floor with the airflow,. Therefore, higher concentrations of PM2 5 gather near the stairway.thirdly, the PM2.5 is easy to gather under the return air outlet due to the suction effect. The average concentration of PM2.5 on the concourse floor when the PSDs are closed is 59.73 μg/m^3^; the average concentration of PM2.5 when the PSDs are opened is 46.67 μg/m^3^. At this time, outdoor air enters the station through the exits, so the concentration of PM2.5 in the four passageways is greater, reaching 57.23 μg/m^3^. Overall, the concentration of PM2.5 in the passageway is close to the simulated concentration with the PSDs opened, while the concentration inside the station concourse is close to the simulated concentration with the PSDs closed. 

For the platform floor, the PM2.5 mainly gathers at the PSDs on both sides. The main reasons for this are, firstly, that pedestrians gather in front of the PSDs to wait for trains; secondly, due to the suction effect, the concentration of PM2.5 under the return outlet is greater. The average PM2.5 concentrations at the platform floor are 60.03 μg/m^3^, 53.59 μg/m^3^, and 79.88 μg/m^3^ for PSDs closed, AB side PSDs opened, and CD side PSDs opened, respectively. When CD side PSDs are opened, the return air outlets are on the opposite side from the CD side PSDs, and the particulate matter tends to settle in the slower airflow area, so the overall concentration is higher.

### 4.3. Particulate Matter Concentration Prediction Based on Surrogate Models

According to Equation (1), the calculation of particulate matter exposure for each pedestrian requires a time-based integral calculation of the particulate matter concentrations along this movement track. Considering that the airflow organization state in the subway station is time-varying, the distribution of particulate matter and its concentration are different when the PSDs are open or closed. How to establish the relationship between the PM2.5 concentration distribution obtained by solving the steady-state DPM model in two airflow organization states and the concentration distribution in the real state so that the time-varying characteristics of the airflow organization state can be considered is the core problem of the accurate calculation of pedestrian exposure. Since there is no definite relationship between the simulated values of particulate matter concentration corresponding to the two simulated states and that of the real state, a data-driven surrogate model between the three is trained by SVR to facilitate the exposure calculation.

Given a training set {(*x_i_*, *y_i_*), …, (*x_N_*, *y_N_*)}, where *x_i_* = [*x_i_*_1_, *x_i_*_2_, …, *x_id_*]^T^, *d* is the dimension, the optimization objective of SVR is to find a regression hyperplane *f*(*x*) = *ω*^T^*ϕ*(*x*) + *b* that minimize of the difference between *f*(*x*) and *y*, which corresponding to the following optimization function [27].

(6) $$\begin{array}{l} \underset{\omega ,b,\xi_{i},\xi_{i}^{*}}{\min}\frac{1}{2}{\left\|\omega \right\|}^{2}+\lambda \sum_{i=1}^{m}(\xi_{i}+\xi_{i}^{*})\\ s.t.f(x_{i})-y_{i}\leq \varepsilon +\xi_{i}\\ y_{i}-f(x_{i})\leq \varepsilon +\xi_{i}^{*}\\ \xi_{i}\geq 0,\;\xi_{i}^{*}\geq 0,\;i=1,2,\ldots ,m \end{array}$$

where *ω* and *b* are the coefficient matrix and the bias matrix, they are used to characterize the regression hyperplane of the SVR model; *ε* is the tolerance error; *ξ*, *ξ* * are slack variables, corresponds to the two error bounds of SVR respectively; *ϕ*(*x*) is the kernel function, when the radial basis function (RBF) is adopted, it satisfies,

(7) $$\kappa (x_{i},x_{k})=\phi {\left(x_{i}\right)}^{T}\phi (x_{k})=\exp(-\sigma {\left\|x_{i}-x_{k}\right\|}^{2})$$

In this study, two hyper-parameters *λ* and *σ* in Equations (6) and (7) are determined using the particle swarm optimization algorithm within the libSVM tool of MATLAB [28].

**Figure 15:**
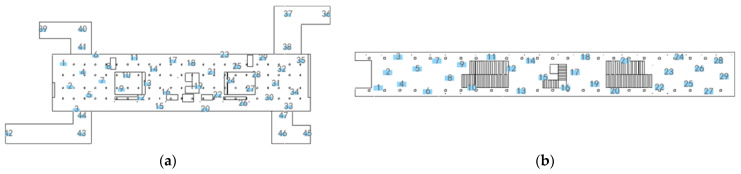
Arrangement of OLHS sampling points: (**a**) Concourse floor; (**b**) Platform floor.

The establishment of the surrogate model is divided into the following two parts: the first step is to conduct a reasonable sampling to obtain the real particulate matter distribution in the station. In this paper, the measurement points are arranged based on the optimal-latin hypercube sampling (OLHS) method [29], this space-filling sampling method can capture the true concentration distribution characteristics of particulate matter with small samples set as fully as possible. Considering the long length of the concourse and platform floor, the space is divided into two parts in length for sampling, and 20 points are sampled in each part using the OLHS method. Besides, three sampling points are arranged in each passageway. After eliminating some sampling points that are not convenient for measurement, the sampling points at the concourse and platform floor are arranged as shown in Figure 15. Considering the limited experimental equipment, a ten-minute sampling was conducted at each point during the period of 9:00–13:00 on 11 November 2020, when the PM2.5 concentration was relatively stable, to obtain the PM2.5 concentration variation and solve for its mean value. The sampling results and the corresponding simulated concentration value at each sampling point are given in Appendix A. 

After the sampling is completed, the second step is to train the surrogate model using the simulated concentration values of the two states as input and the measured concentration values as output. 

**Figure 16:**
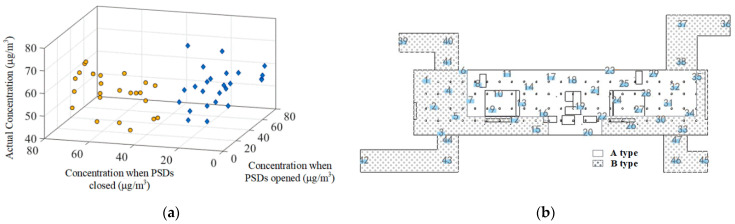
Cluster and spatial region classification results: (**a**) Cluster result; (**b**) Concourse floor areas corresponding to different types of sampling points.

For the concourse floor, there are two main particulate matter sources, the PM2.5 within the supply air, and the outdoor PM2.5 entering from the passageway when the PSDs are opened. The two sources cause the PM2.5 concentration data at the sampling points to show different distribution characteristics. To train surrogate models with better generalization ability, the *K*-means algorithm is used to classify the sampling data at the concourse floor into two categories. Figure 16 shows the classification results. Combined with the CFD simulation results, it can be seen that the A-type point is more influenced by the air supply pollution source; the B-type point is more influenced by external pollution sources. Surrogate models are built for the sampling points of types A and B, respectively. Since the regression effect of surrogate models is limited by the amount of data in the training set, to ensure a high generalization ability, the training set is set to be larger, with the test set data accounting for 10% to 20% of the total samples. Type A has 23 sampling points; 20 sets of data are randomly taken as the training set and three sets of data as the test set. Type B has 24 sampling points, 20 sets of data are randomly selected as the training set, and four sets of data as the test set. Later, when calculating the exposure, the data points located in the different areas will use the corresponding surrogate model according to the division results of the concourse floor shown in Figure 16b.

**Figure 17:**
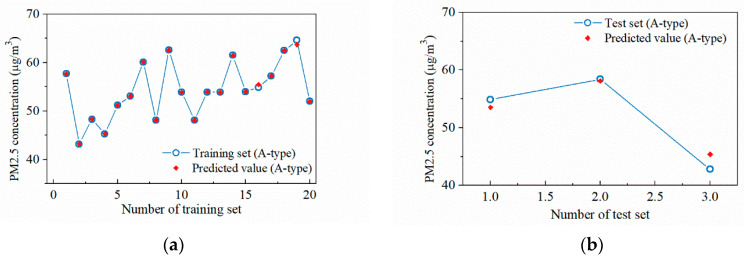
Regression result of the A-type sampling points at the concourse floor: (**a**) Training set; (**b**) Test set.

The surrogate model training results for A-type sampling points are shown in Figure 17a,b, with the optimal hyper-parameter combination of [*λ*, σ] = [15.2, 0.03]. The determination coefficient *R*^2^ is used to characterize the regression result [29], as shown in Equation (8). the closer *R*^2^ is to 1, the better the regression effect, an *R*^2^ value of 0.995 for the training set and 0.988 for the test set.

(8) $$R^{2}=1-\sum_{i=1}^{n}(y_{i}-{\hat{y}}_{i}{)}^{2}/\sum_{i=1}^{n}(y_{i}-\bar{y}{)}^{2}$$

where *y_i_*, $\bar{y}$, $\hat{y}$*_i_* denote the measured data, the mean value of the measured data, and the predicted data, respectively, and *n* is the number of data.

**Figure 18:**
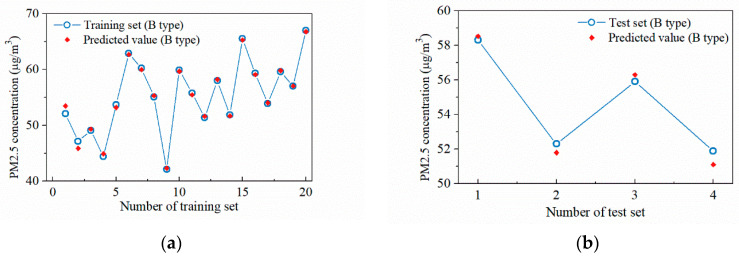
Regression result of the B-type sampling points at the concourse floor: (**a**) Training set; (**b**) Test set.

The surrogate model training results for B-type sampling points are shown in Figure 18a,b, with the optimal hyper-parameter combination of [*λ*, σ] = [907.0, 0.09]. An *R*^2^ value of 0.989 for the training set and 0.992 for the test set.

**Figure 19:**
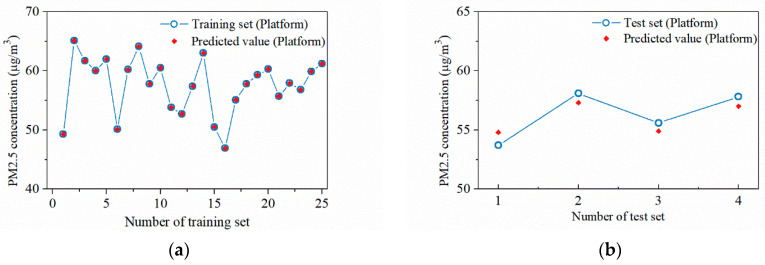
Regression result of the sampling points at the platform floor: (**a**) Training set; (**b**) Test set.

Unlike the concourse floor, the main particulate matter source of the platform floor is only the PM2.5 within the supply air, so its surrogate model can be trained without clustering the data. Among the 29 sets of data at the platform floor, 25 sets of data are randomly selected as the training set and four sets of data are used as the test set. The particulate concentration values at the sampling points under the three conditions of PSDs closed, AB side PSDs open and CD side PSDs open are used as the input, and the measured results are used as the output; the training of the surrogate model is conducted, as shown in Figure 19, with the optimal hyper-parameter combination of [*λ*, σ] = [677.6, 2.90]. The *R*^2^ value for the training set is 0.998 while the *R*^2^ value for the test set is 0.932.

### 4.4. Exposure Calculation

**Figure 20:**
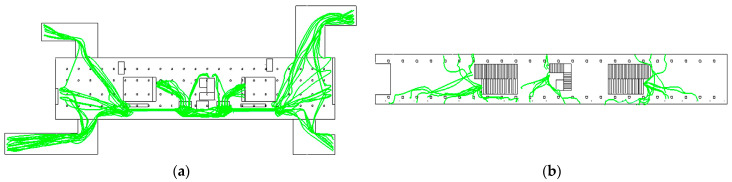
The streamlines of 50 boarding pedestrians: (**a**) Concourse floor; (**b**) Platform floor.

In calculating the exposure, the pedestrian streamlines from Massmotion software are imported into Fluent to obtain the simulated values of particulate matter concentration at each point on the pedestrian streamlines. Fifty samples are randomly selected among the boarding pedestrians and the alighting pedestrians, respectively, where the streamlines of the 50 boarding pedestrians are shown in Figure 20.

**Figure 21:**
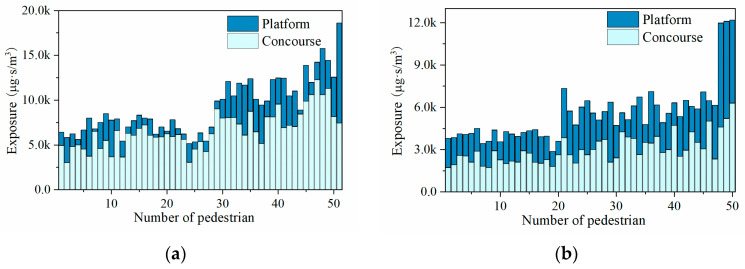
PM2.5 exposure calculation results: (**a**) Boarding; (**b**) Alighting.

Furthermore, the exposure of pedestrians during the boarding and alighting process can be calculated using the surrogate model. The specific process of exposure calculation can be briefly expressed as follows. Firstly, considering that the sampling interval of the instrument used for field measurements is 1 s, for each pedestrian, their position is obtained once every second according to the PFA results. Then, the PM2.5 concentration is calculated based on the surrogate model corresponding to that location. Finally, this concentration value is regarded as the average exposure concentration of the pedestrian to PM2.5 in this second, and then, according to Equation (1), the exposure concentration of the pedestrian in the subway station space is calculated cumulatively to obtain the exposure amount. The exposure calculation results for the sampled 50 pedestrians are shown in Figure 21. There are individual differences in the calculated exposures due to factors such as pace speed, route choice, and train waiting time. The average particulate matter exposure of pedestrians during the boarding process is 9985.74 μg·s/m^3^, and the average particulate matter exposure of pedestrians during the alighting process is 5761.48 μg·s/m^3^. Due to the need to queue for security checks and swipe cards to enter the station, the particulate exposure at the concourse floor during the boarding process is much higher than at the platform floor. The amount of particulate matter exposure at the concourse floor is comparable to that at the platform floor during the alighting process. The particulate matter exposure of pedestrians during the boarding process is about twice as much as that of the alighting process.

## 5. Validation and Comparison

### 5.1. Experimental Validation

The experimental results are used to validate the reasonableness of the particulate matter concentration simulations and exposure calculations. 

#### 5.1.1. CFD Simulation Results Validation

**Figure 22:**
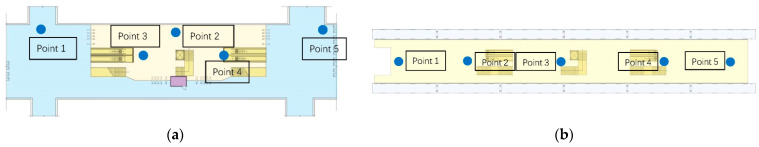
Arrangement of measurement points at two floors: (**a**) Concourse floor; (**b**) Platform floor.

To validate the CFD model simulation results, the actual PM2.5 concentrations in the subway stations were measured, and the measurement points at the concourse floor and platform floor were arranged as shown in Figure 22. The measurements were conducted during the period of 9:30–11:30 on three weekdays (11 to 13 November 2020) when the outdoor particulate matter concentrations were almost the same, and each measurement point was measured for 60 min to obtain the mean PM2.5 concentration. Table 5 gives the measurement results.

**Figure 23:**
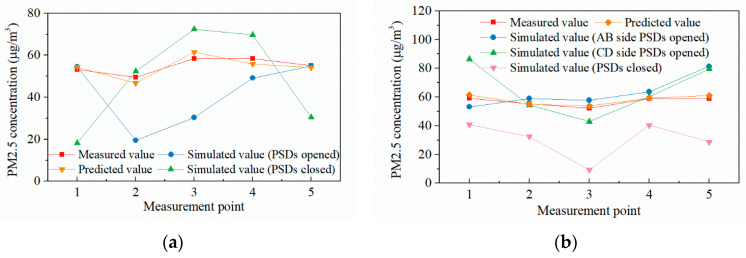
Comparison of PM2.5 concentration simulated results with measured results at measurement points: (**a**) Concourse floor; (**b**) Platform floor.

The total mean PM2.5 concentrations of the concourse and platform floors are 54.91 μg/m^3^ and 57.02 μg/m^3^. Figure 23 shows the comparison of simulated, measured, and predicted values, the predicted results fit well with the measured results, which validated the high accuracy of surrogate models. Meanwhile, the measurement results are within several simulated state values, the reasonableness of the simulation method is verified. 

#### 5.1.2. Exposure Calculation Results Validation

In addition, to verify the accuracy of the pedestrian exposure calculation, mobile sampling observations of the particulate matter exposure during the boarding and alighting processes were conducted. The specific process of mobile sampling has two parts in the boarding process, the experimenter holds the portable measurement device Sinffer4D, enters the concourse floor from the entrances, passes through the security checkpoint and the automatic ticket machine, and then independently selects the stairs or escalator down to the platform floor and waits for the vehicle to enter the station; in the alighting process, after the train enters the platform, the experimenter departs from a random PSD, independently selects the stairs or escalator up to the concourse floor, passes through the automatic ticket machine and then goes to the exit.

**Figure 24:**
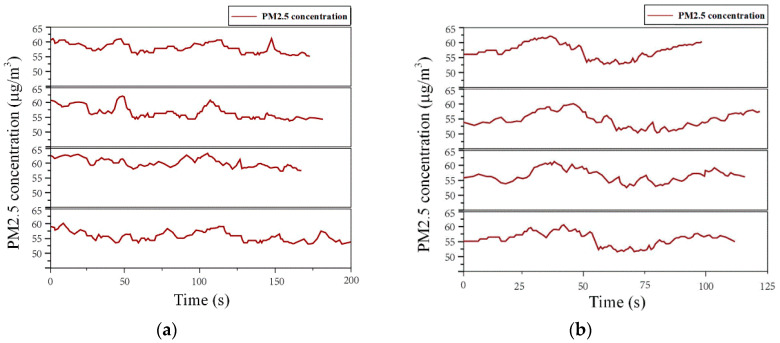
PM2.5 concentration-time curves for pedestrians during boarding and alighting: (**a**) Boarding; (**b**) Alighting.

For the boarding process, in each round of the experiment, four experimenters entered from four entrances of A, B, C, and D to obtain PM2.5 time-concentration curves of the boarding process. This experiment was conducted in five rounds, and 4 × 5 = 20 sets of PM2.5 time-concentration curves could be obtained. The sampling of the alighting process was similar to that of the boarding process, with the experimenters traveling from the PSDs to the four exits: A, B, C, and D. The experiment was conducted with two 25-year-old males and two 25-year-old females, with normal and stable pace speed. Figure 24a,b show the PM2.5 time-concentration curves obtained by the experimenter in one of the experimental rounds for the boarding process and the alighting process, respectively.

During the sampling process, the instrument recorded data every 1 s, and then the pedestrian PM2.5 exposure of the boarding and alighting process can be calculated according to Equation (1). The average value is calculated for 20 sets of data, and the results are shown in Table 6, where the average exposure concentration is the ratio of the average PM2.5 exposure to the average time.

**Figure 25:**
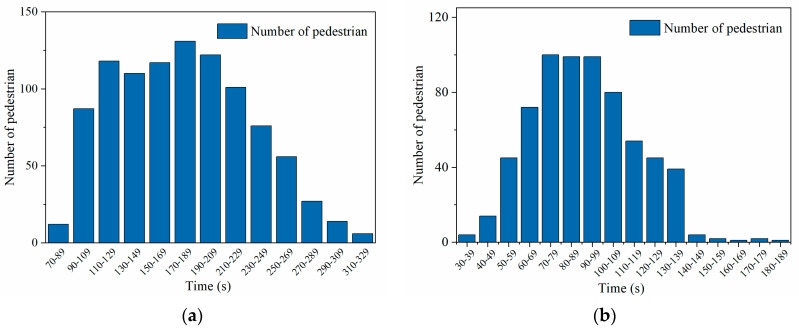
Residence time distribution of boarding and alighting pedestrians: (**a**) Boarding; (**b**) Alighting.

Figure 25 shows the residence time distribution of boarding and alighting pedestrians in the subway station space obtained from the PFA simulation. The minimum value of the total time required for boarding is 50 s, the maximum value is 450 s, and the average value is 157.2 s. The relative error with the measured average value of 170.1 s for the boarding process is 9.24%; the minimum value of the total time required for alighting is 33 s, the maximum value is 180 s, and the average value is 92.3 s. The relative error with the measured average value of 103.4 s for the alighting process is 10.7%. Overall, the error between the simulated and measured values is about 10%, which verifies the PFA simulation.

For the PM2.5 exposure value, the relative error between the calculated value of 9985.74 μg·s/m^3^ and the measured value of 10,601.3 μg·s/m^3^ for the boarding process is 5.80%, and the relative error between the calculated value of 5761.48 μg·s/m^3^ and the measured value of 6057.8 μg·s/m^3^ for the alighting process is 4.89%, which proved the accuracy of the proposed exposure calculation method. The calculated values are smaller than the measured values, which may be attributed to the fact that the dust generated during pedestrian walking is neglected in the CFD simulation, so it is necessary to take this part of the particulate matter generation source into account in the subsequent study as well.

### 5.2. Comparison of Different Exposure Calculation Methods

Since the relevant literature usually uses a simplified Equation (2) for exposure calculation, i.e., using the average pollutant concentration within each microenvironment and the residence time of a person in this microenvironment. To illustrate the advantages of the proposed method in terms of calculation accuracy, the two methods are compared.

The concourse floor and platform floor of the subway station are regarded as two microenvironments. The average concentration of particulate matter is obtained by the CFD model, and the residence time of boarding and alighting pedestrians in the two microenvironments is obtained by the PFA model so that the PM2.5 exposure of pedestrians in different microenvironments for different behavioral purposes can be obtained as Table 7, in which the average PM2.5 concentrations is characterized as the mean value of the spatially averaged concentration in multiple airflow organization states.

Based on the results in Table 7, when the average PM2.5 concentration and average time are used for calculation, the PM2.5 exposure for the pedestrian boarding process is 5577.5 + 3377.3 = 8954.8 μg·s/m^3^, and the exposure for the exiting process is 2731.8 + 2641.3 = 5373.1 μg·s/m^3^. If the method proposed in this paper is called the “integration method” and the above method is called the “average concentration method”, the relative errors between their calculated results and the actual measured values are shown in Table 8. In the last column of Table 8, the first value indicates the relative error between the calculated exposure of the boarding process using the corresponding method and the measured exposure, and the second value indicates the relative error between the calculated exposure of the alighting process using the corresponding method and the measured exposure.

As can be seen from Table 8, since the integration method has a much smaller time scale and can accurately consider the exposure path of each pedestrian, its error is much smaller compared to the average concentration method, and the exposure calculation error of the average concentration method for the boarding and alighting process is 2.7 times and 2.3 times higher than that of the integration method, respectively.

Although the integration method in this paper has higher accuracy compared to the average concentration method, its computational complexity is also higher, so it is necessary to further discuss the application occasions of both methods. The average concentration method does not require field measurements to form the surrogate model of simulated particulate matter concentrations and actual concentrations, boundary conditions of the CFD model and the PFA model can be given according to the external environment and estimated passenger flow of the subway station. Therefore, it is more suitable for pollutant exposure assessment at the early stage of station design to help adjust ventilation system parameters or for comprehensive design optimization taking into account public health issues. The integral method relies on actual measurement data, its advantages lie in the high computational accuracy and the ability to accurately take into account the behavioral characteristics of pedestrians, making it more suitable for accurate exposure assessment of established subway stations to analyze the impact of pollutants on population health risks.

## 6. Main Points and Limitations

For the particulate matter exposure assessment and accurate exposure calculation of pedestrians in underground subway stations, this paper proposes a new method combining numerical simulation and actual measurement. The innovative points of the proposed method can be listed as follows.

(1) From the four perspectives of the air environment, the space utilization rate of the station platform, the airflow organization mode, and the matching degree between the designed and actual passenger flow, this paper selects suitable station characteristics to form a subway station classification method adapted to carry out the exposure assessment study. The final selected underground subway station with an island platform and the PSD system is representative of Wuhan subway stations, which account for 76.2% of the total number of stations; it is also one of the main subway station construction types across China. This ensures the generality and adaptability of the research methods and conclusions to the same type of stations and to the research objectives.
(2) This paper combines PFA with CFD models to provide a new quantitative method for exposure assessment. PFA not only helps to determine the location and value of the pedestrian particulate matter generation sources, but also to obtain the trajectory of pedestrians, which helps to more accurately analyze the impact of particulate matter on each pedestrian. In future studies, the specific effects of different types of pollutants on specific populations, such as elderly people with slower travel speeds, could be further analyzed.
(3) In this paper, the complex correlation between the simulated spatial concentration distribution of PM2.5 under several different airflow organization states and the actual PM2.5 concentration distribution is established by the machine learning method, which takes into account the time-varying characteristics of the airflow organization states, thus eliminating the need for a complex CFD numerical simulation of transient fields and facilitating the accurate calculation of exposure.

From the application perspective, the proposed method contains the following limitations.

(1) To simplify the simulation model, the air leakage when the PSDs are closed is ignored in the CFD simulation; meanwhile, the particulate matter entering the platform from the tunnel and carriages when the PSDs are opened is also neglected. Thus, the particulate matter generated by the train operation is not appropriately considered, which results in the simulated concentration on the platform being lower than the actual measured value, which causes further errors in the exposure calculation. To further improve the calculation accuracy, it is necessary to build a more refined simulation model.
(2) Due to the limitation of field measurements, the simulated and measured data in this paper are obtained during the off-peak hours. However, if in the peak hours, on the one hand, the field measurement will be more difficult and the concentration sampling will be more easily disturbed by external factors; on the other hand, the resuspension of particulate matter caused by pedestrians walking may no longer be negligible because the number of pedestrians becomes larger, which may bring new particulate matter sources. Therefore, the applicability of the proposed method during peak hours needs to be investigated further.

## 7. Conclusions

Combining PFA and CFD analysis, this paper presents a new method to perform the fine particulate matter exposure assessment in an underground subway station, and the following conclusions can be drawn:

(1) To improve the generality of the research methods and conclusions and to adapt to the pollutant exposure assessment study, suitable subway station characteristics are selected to classify the stations in Wuhan. The underground subway station with the PSD system and an island platform is selected as the type of station to be studied. Finally, the Sports Center Station of Line 3, where there is a high mismatch between the design passenger flow and the actual passenger flow, is studied as an example.
(2) The trajectory of pedestrians in the subway station has a large influence on the spatial concentration distribution characteristics of particulate matter and determines the exposure flow lines. Taking the PFA results into account when performing numerical simulations and exposure calculations can help improve the accuracy of quantitative analysis.
(3) The spatial concentration distribution of particulate matter in subway stations varies greatly under different airflow organization methods, so the exposure assessment by only one airflow organization method will have large errors. The surrogate model established by using simulated data and actual measurement data can not only consider the spatial concentration distribution of particulate matter under various airflow organization conditions, but also make the calculation process of the exposure amount easier.
(4) The smaller the time scale used for the exposure calculation, the more accurate the results obtained. The calculation method proposed in this paper takes 1 s as the calculation step, and the relative error with the real exposure is only about 5%, which is much smaller than that of the average concentration method.

## Figures and Tables

**Table 1:** Statistics on the behavior of pedestrians entering the subway station during one off-peak hour in the morning and the afternoon.

	AM 10:30–11:30		PM 15:00–16:00	
Exit	Ticket	Card	Ticket	Card
A	206	829	121	479
B	117	423	127	463
C	71	277	55	228
D	203	806	98	407

**Table 2:** Statistics of pedestrians’ information during one weekday morning off-peak hour.

	Boarding	Alighting
Upward (CD side)	2143	951
Downward (AB side)	789	918

**Table 3:** Boundary conditions for the CFD model.

Boundary Condition	Value	Boundary Condition	Value
*v_c_*	2.3 m/s	*μ_out_*	61.5 μg/m^3^
*v_p_*	1.9 m/s	*μ_sup_*	24.4 μg/m^3^
*W_l_*	20 W/m^2^	*r_per_*	10 mg/(person·h)
*ρ_p_*	1050 kg/m^3^	*r_eq_*	3.78 × 10^−9^ kg/s
*D*_p_	2.5 × 10^−6^ m		

**Table 4:** IATA Waiting Level Table.

Service Level	Density (Person/m^2^)	Color Representation
A	*x* ≤ 0.370	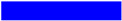
B	0.370 < *x* ≤ 0.435	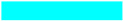
C	0.435 < *x* ≤ 0.526	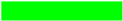
D	0.526 < *x* ≤ 0.667	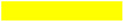
E	0.667 < *x* ≤ 1	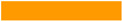
F	1 < *x*	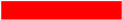

**Table 5:** Actual PM2.5 Concentration Measurement Results (Unit: μg/m^3^).

	Concourse Floor					Platform Floor				
Point	1	2	3	4	5	1	2	3	4	5
Wed.	51.13	47.05	59.02	61.03	56.02	58.12	56.32	52.32	60.02	59.32
Thur.	52.22	50.88	57.14	56.15	54.56	58.78	53.55	51.88	59.13	58.87
Fri.	56.30	50.43	58.80	58.12	54.77	60.99	55.80	53.98	58.54	58.94
Mean	53.22	49.45	58.32	58.42	55.12	59.29	55.22	52.33	59.23	59.04
	54.91					57.02				

**Table 6:** PM2.5 Exposure Measurement Results.

	Average Exposure	Average Time	Average Concentration
Boarding	10,601.3 μg·s/m^3^	170.1 s	59.6 μg/m^3^
Alighting	6057.8 μg·s/m^3^	103.4 s	55.4 μg/m^3^

**Table 7:** PM2.5 Exposure Calculation Results Using Average Concentration.

	Average Concentration	Boarding Residence Time	Alighting Residence Time	Exposure Value (μg·s/m^3^)
Concourse	53.2 μg/m^3^	104.84 s	51.35 s	5577.5, 2731.8
Platform	64.5 μg/m^3^	52.36 s	40.95 s	3377.2, 2641.3

**Table 8:** PM2.5 Exposure calculation results and errors of different methods.

	Boarding	Alighting	Relative Errors (%)
Measured Exposure	10,601.3 μg·s/m^3^	6057.8 μg·s/m^3^	
Integration method	9985.7 μg·s/m^3^	5761.5 μg·s/m^3^	5.8, 4.9
Average concentration method	8954.8 μg·s/m^3^	5373.1 μg·s/m^3^	15.5, 11.3

## Data Availability

The measured and experimental data involved in this paper are the work results of the research team, and relevant data have been listed in the article or Appendix A.

## Appendix A

**Table A1:** Measured and simulated PM2.5 concentrations at sampling points of the concourse floor.

**Point**	**1**	**2**	**3**	**4**	**5**	**6**	**7**	**8**
Measured	42.1	47.1	52.1	51.9	51.4	55.9	60.1	48.3
PSDs opened	41.747	49.406	54.625	54.054	58.496	55.927	39.561	45.777
PSDs closed	19.550	21.734	25.302	36.364	34.610	13.315	69.836	48.175
**Point**	**9**	**10**	**11**	**12**	**13**	**14**	**15**	**16**
Measured	57.2	62.5	43.6	65.5	62.6	48.1	67.0	52.0
PSDs opened	17.896	46.609	10.966	75.272	30.254	4.431	72.876	42.699
PSDs closed	71.835	75.709	44.729	22.722	73.201	57.684	37.309	53.960
**Point**	**17**	**18**	**19**	**20**	**21**	**22**	**23**	**24**
Measured	43.2	45.3	53.9	54.9	57.7	48.1	51.2	53.9
PSDs opened	34.967	16.009	23.636	26.811	33.221	48.221	46.418	42.105
PSDs closed	41.326	51.133	62.022	62.893	64.417	12.026	52.635	68.065
**Point**	**25**	**26**	**27**	**28**	**29**	**30**	**31**	**32**
Measured	64.6	51.9	61.5	59.0	54.8	55.8	53.9	53.1
PSDs opened	42.000	52.466	23.514	41.85	53.156	55.788	38.387	58.309
PSDs closed	73.779	23.678	73.039	67.183	59.872	23.710	57.630	51.894
**Point**	**33**	**34**	**35**	**36**	**37**	**38**	**39**	**40**
Measured	60.2	54.0	44.4	58.0	57.0	53.7	62.9	59.3
PSDs opened	65.817	57.914	50.091	58.005	56.958	53.757	62.963	59.366
PSDs closed	28.433	35.178	29.615	0.000	22.471	28.396	0.000	16.15
**Point**	**41**	**42**	**43**	**44**	**45**	**46**	**47**	
Measured	52.3	58.3	55.1	49.1	59.6	59.9	53.9	
PSDs opened	52.263	58.313	55.109	49.021	59.588	59.859	53.817	
PSDs closed	17.053	0.000	16.009	79.739	0.000	10.803	14.638	

**Table A2:** Measured and simulated PM2.5 concentrations at sampling points of the platform floor.

**Point**	**1**	**2**	**3**	**4**	**5**	**6**	**7**	**8**
Measured	46.9	61.7	65.1	49.3	59.9	53.7	62.0	55.1
AB PSDs opened	31.893	53.157	36.923	48.200	37.013	44.593	55.492	58.844
CD PSDs opened	47.613	86.534	68.361	46.525	89.176	53.04	76.636	54.417
PSDs closed	31.623	40.377	74.072	45.330	34.944	52.091	52.625	32.486
**Point**	**9**	**10**	**11**	**12**	**13**	**14**	**15**	**16**
Measured	58.1	56.8	57.4	57.8	57.8	63.0	53.8	52.7
AB PSDs opened	69.300	62.578	57.209	39.569	58.178	18.526	57.875	38.695
CD PSDs opened	49.300	51.500	55.204	80.187	41.508	96.757	43.071	58.488
PSDs closed	30.030	23.732	51.545	34.690	29.726	29.400	6.350	27.242
**Point**	**17**	**18**	**19**	**20**	**21**	**22**	**23**	**24**
Measured	50.1	55.7	57.8	50.5	60.0	60.5	60.3	64.1
AB PSDs opened	43.036	42.133	48.513	56.821	64.933	31.702	64.841	70.305
CD PSDs opened	52.294	89.515	88.839	28.158	84.681	50.728	63.499	68.810
PSDs closed	26.465	10.405	46.501	13.850	15.620	75.781	39.223	39.779
**Point**	**25**	**26**	**27**	**28**	**29**			
Measured	57.9	55.6	60.2	59.3	61.2			
AB PSDs opened	42.899	42.020	25.109	25.106	82.000			
CD PSDs opened	38.339	59.350	39.234	90.801	79.971			
PSDs closed	61.663	35.621	91.315	40.550	26.605			
